# Successful Propranolol Treatment of a Large Size Infantile Hemangioma of the Face Causing Recurrent Bleeding and Visual Field Disruption

**Published:** 2015-01

**Authors:** Muhammad Saaiq, Bushra Ashraf, Saad Siddiqui, Shehzad Ahmad, Muhammad Salman Zaib

**Affiliations:** Department of Plastic Surgery and Burns, Pakistan Institute of Medical Sciences (PIMS), Shaheed Zulfiqar Ali Bhutto Medical University (SZABMU), Islamabad, Pakistan

**Keywords:** Infantile hemangioma, Bleeding, Visual field, Propranolol

## Abstract

A 29 days old Pakistani female infant was presented to our outpatient department with two weeks history of a rapidly progressing large size facial hemangioma involving most of the right cheek and right eyelids. The infant was unable to open the right eye. There was also a small hemangioma on the right second toe. Additionally, three similar lesions were found on the right side of the palate and adjoining buccogingival surfaces. The parents were particularly concerned about the explosive progression of the lesions, recurrent bleeding episodes from ulcerated areas of the cheek lesion and complete occlusion of the right eye. Following four weeks therapy with propranolol in a dose of 2 mg/kg/day, the hemangiomas rapidly regressed, the bleeding episodes ceased and the infant started opening the eye.

## INTRODUCTION

Infantile hemangiomas constitute the commonest benign vascular neoplasms of infancy affecting 4-10% of the full-term infants of white race and are more frequent among premature, female babies of low birth weight.^1^^,^^2^ Their natural history typically follows a cycle consisting of proliferation, involution and involuted phases. They usually manifest within the first 2 months of life and proliferate rapidly during the first year. The lesions then start spontaneous involution, so that 50% are involuted by the age 5 and 70% by age 7.^1^^-^^3^

Although benign, and known to undergo spontaneously regression, the anatomic location and variable growth patterns of hemangiomas will lead to serious functional or cosmetic concerns in up to 10% of the infants. Among them, judicious early intervention is needed to address the issues such as visual field disruption, respiratory obstruction, congestive heart failure, severe hemorrhage, or serious disfigurement.^4^^,^^5^

Despite the frequency of these vascular neoplasms of infancy, their most ideal management continues to be explored. Globally there has been a recent recognition of the efficacy of propranolol in managing these neoplasms. To the best of our knowledge, ours is the first case to be reported in this regard from any plastic surgical facility from Pakistan. This uniqueness prompts us to share our experience.

## CASE REPORT

**Fig. 1:**
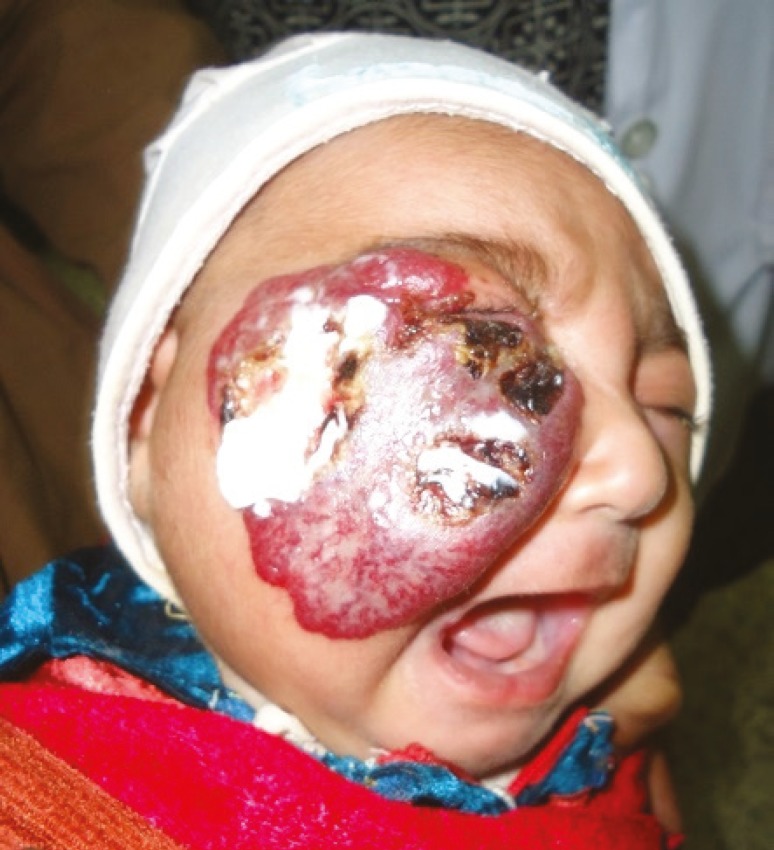
The infant at initial presentation with extensive hemangioma involving most of the right cheek and right eyelids. There were five ulcerated, hemorrhagic spots which had been covered with dressings by the parents.

**Fig. 2:**
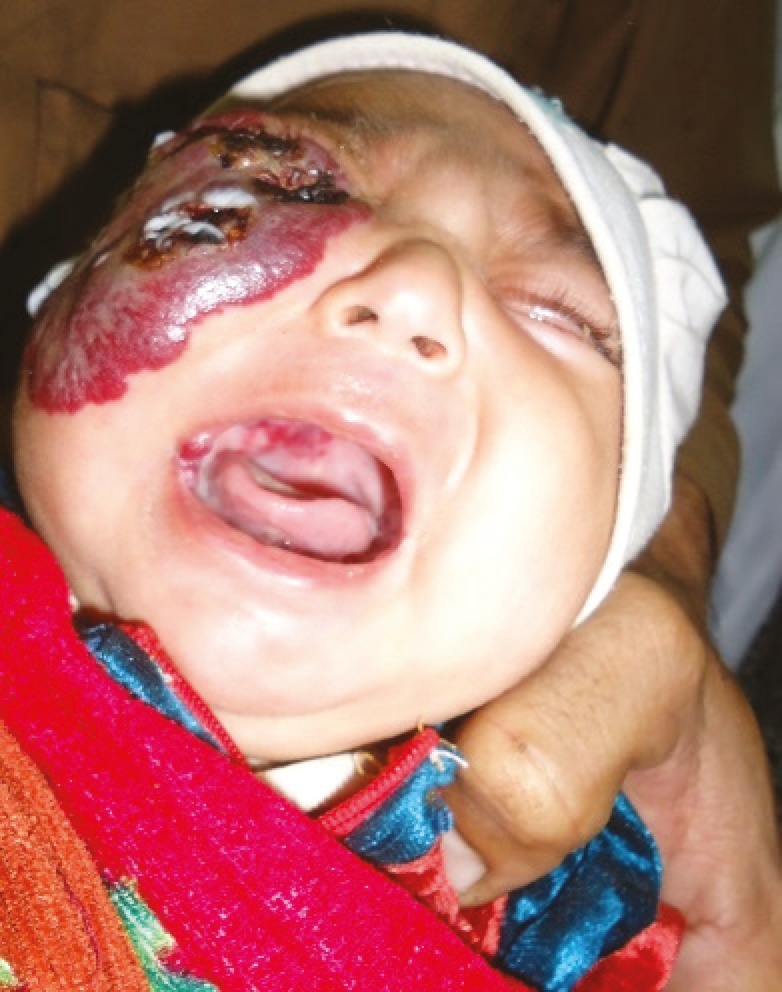
Three hemangiomatous lesions on the right side of palate and adjacent buccogingival surfaces could be also be visualized as the child cried.

**Fig. 3:**
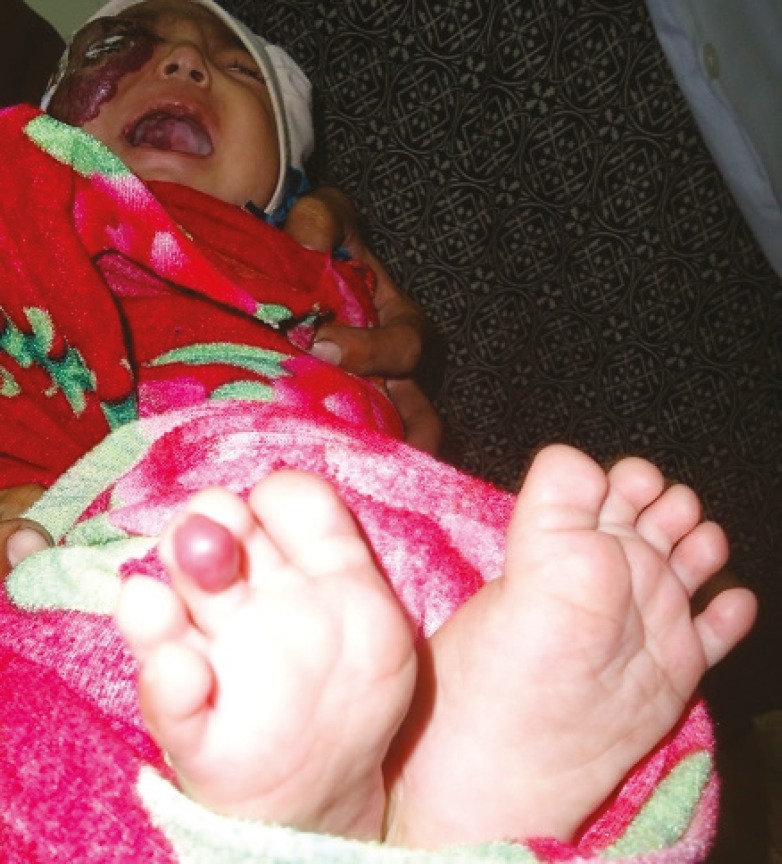
Small hemangioma was also present on the right third toe.

A 29 days old Pakistani female infant was brought by parents to our outpatient department with two weeks history of a rapidly progressing purplish color, raised cutaneous lesion with bosselated surface involving most of the right cheek and right eyelids with associated inability to open the right eye. (Figure 1). The parents were particularly concerned about the explosive progression of the lesion on the face, recurrent bleeding episodes from ulcerations and complete occlusion of the eye on the affected side. Clinical examination of the infant revealed features suggestive of a large hemangioma on the right face (Figure 1), three similar hemangiomas on the right side of the palate and adjoining buccogingival surfaces (Figure 2), and a small hemangioma on the right third toe (Figure 3). Ulcerated areas were noticed on the hemangioma of the cheek. The right eye could not be opened.

The systemic physical examination of the infant, laboratory work up, chest x-ray, and ultrasonography of the abdomen were all unremarkable. Ophthalmology colleagues were consulted to rule out any associated eye abnormalities. The infant did not have any features suggestive of the PHACES syndrome (i.e. posterior fossa malformations, hemangioma of the cervicofacial region, arterial anomalies, cardiac anomalies, eye abnormalities and sterna defects). Family history of atopy or any history of recurrent wheezing in the infant was sought to rule out any possible contraindications to propranolol treatment.

Written informed consent was taken from the parents of the infant for instituting propranolol treatment and taking serial photographs through the course of the therapy. Prior to initiating propranolol therapy, pretreatment cardiovascular evaluation of the infant was undertaken to rule out any contraindications to propranolol therapy. The evaluation was performed by our cardiology colleagues and included baseline clinical observation of pulse, blood pressure and respiratory rate, ECG recording and echocardiographic assessment of the infant. All these were within normal limits in our infant. 

**Fig. 4:**
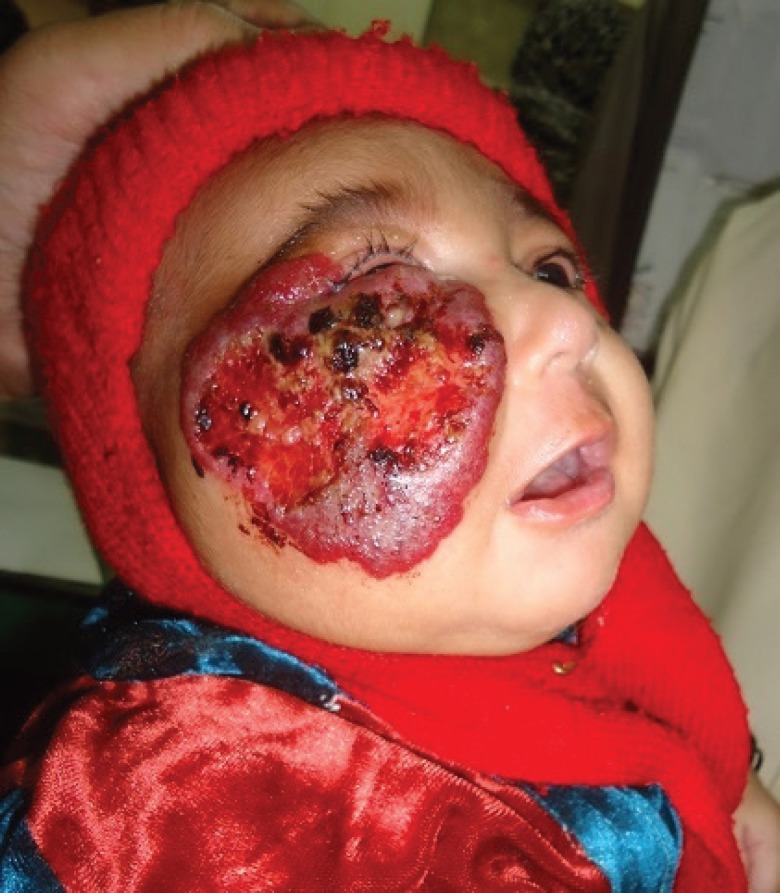
Three days after initiation of the propranolol therapy, there was visible alteration in the color of the lesion, softening in texture and the infant could slightly open the right eye.

For oral administration of the first dose of propranolol, we kept the infant under observation/monitoring for four hours in the hospital. The initial loading dose we employed was one third of the calculated 2 mg/kg/ day dose of propranolol. The mother was encouraged to continue breast feeding. The post dose pulse and blood pressure measurements were carried out every 15-20 minutes for the first four hours, and they were all stable in our infant. The infant was subsequently sent home with advice to mother regarding adherence to appropriate dosage regimen and a follow up visit after 72 hours (Figure 4). 

**Fig. 5:**
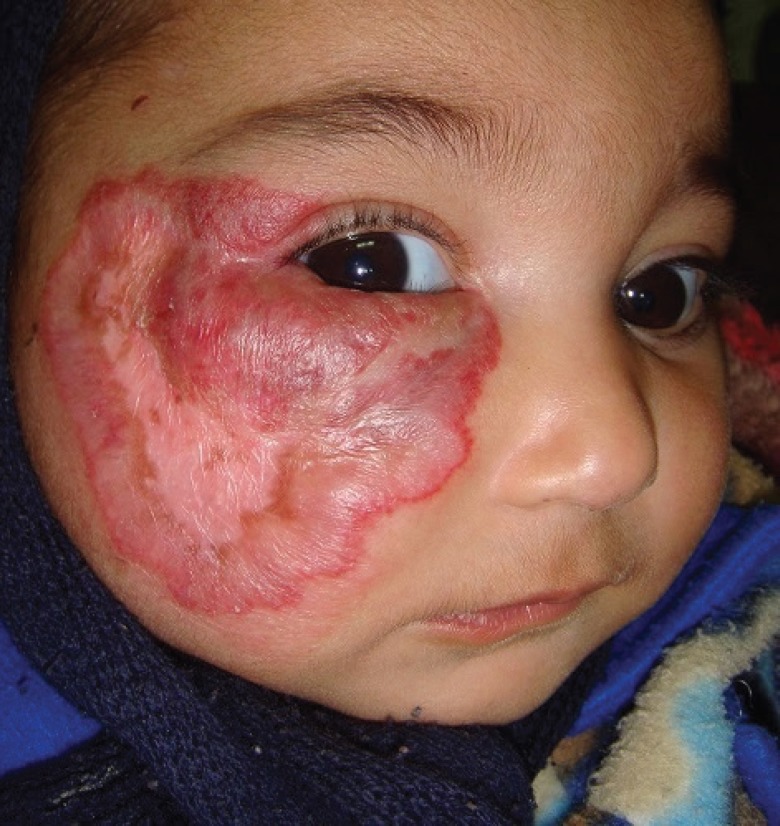
There was considerable improvement after completing four weeks therapy.

From then on, the infant was followed up on outdoor basis every fortnightly and response to therapy was recorded through serial photographs. The mother was educated to notice and report any side effects of the therapy. The treatment was stopped after four weeks when there was a sustained halt in the growth of the hemangiomas (Figure 5).

## DISCUSSION

We observed swift and considerable improvement in the otherwise alarmingly growing hemangiomas in our infant. Léauté-Labrèze *et al.*^6^ from France were pioneer in work on propranolol’s efficacy in managing infantile hemangiomas. They initially observed rapid regression of an infantile hemangioma while treating an infant for the associated obstructive hypertrophic myocardiopathy. The exact mechanism that underlies this magical effect still continues to be sought. The possible mechanisms involved include induction of vasoconstriction and hypoxia, up-regulation of cellular apoptosis of capillary endothelial cells, and down regulation of angiogenic factors such as the basic fibroblast growth factor (bFGF) and vascular endothelial growth factor (VEGF). Propranolol has also been reported to be a selective inhibitor of matrix metalloproteinase IX.^6^^-^^8^

In our infant, we employed propranolol in a dose of 2 mg/kg/day in three divided doses for four week weeks. In most of the published studies, a dosage regimen of 2 mg/kg/day, divided in doses of 3-4 times daily has been reported. The reported duration of therapy ranges from 4 weeks to 12 months depending on the variably reported response rate.^9^^,^^10^ Generally accepted consensus on the ideal treatment regimen with propranolol has yet to be evolved. 

In our infant, we did not encounter any serious untoward side effect that could dictate discontinuation of the therapy. Our experience conforms to most of the published literature where safety of propranolol in infants has been well established. Propranolol has been used in infants for a variety of medical conditions in doses as high as 7 mg/kg/day without any serious side effects. No mortality has been documented to date. The potential adverse effects include transient bradycardia, hypotension, hypoglycemia, bronchospasm and gastrointestinal discomfort.^10^^-^^13^

The transient bradycardia and hypotension warrant close monitoring at the onset of the treatment. The bronchospasm is usually seen as an exacerbation among those infants who have underlying reactive airways, hence warranting seeking any family history of atopy or recurrent wheezing before instituting the therapy. As the β-blockers decrease lipolysis, glycogenolysis, and gluconeogenesis, the infant can develop hypoglycemia. The hypoglycemic symptoms are additionally masked by the β-blockade. The first week of neonatal life is most crucial as the neonate gradually reaches his optimal milk intake and spontaneous hypoglycemia is more likely to develop, hence β-blockers should be avoided during this period.^12^^-^^14^

In case of spontaneously involuting hemangiomas, half of the children do have some residual cosmetic deformity that will need surgical interventions in their subsequent adult life. There could be some degrees of scarring, a fibrofatty residuum, cutaneous blemish, yellowish hypoelastic patches or crepelike laxity of the affected area.^1^^-^^4^ Similarly, propranolol therapy does not have a fairy-tale ending as far as the cosmetic appearance of the affected area is concerned. We need to have long term follow up studies to assess as to what percentage of propranolol treated infants need cosmetic corrective surgeries as compared to those who are managed with other modalities including watchful wait for spontaneous involution.

It was shown that propranolol in a dose of 2 mg/kg/day in three divided doses is a safe and effective first line therapy for effecting rapid regression of large hemangiomas that are otherwise blocking the visual field and carry a risk of causing deprivational amblyopia in an infant. 

## Note

Please cite this paper as:

Saaiq M, Ashraf B, Siddiqui S, Ahmad S, Zaib MS. Successful Propranolol Treatment of a Large Size Infantile Hemangioma of the Face Causing Recurrent Bleeding and Visual Field Disruption. World J Plast Surg 2015;4(1):79-83.
